# Quality of Care in Pediatric Palliative Care: A Scoping Review

**DOI:** 10.3390/children10121922

**Published:** 2023-12-13

**Authors:** Tania Ruiz-Gil, Francisco Ródenas-Rigla

**Affiliations:** Polibienestar Research Institute, University of Valencia, 46022 Valencia, Spain; francisco.rodenas@uv.es

**Keywords:** pediatric palliative care, Spain, quality of care, scoping review

## Abstract

Palliative care (PC) enhances the quality of life for patients and families facing life-threatening conditions. While PC is well-established for adults, not all practices apply to pediatrics. Consequently, specific quality indicators for Pediatric Palliative Care (PPC) must be identified. This scoping review aimed to identify the variables associated with the quality of care of PPC patients in Spain, focusing specifically on three areas: structure and process of care, psychological aspects of care, and care of patients approaching the end of life. The review was conducted following PRISMA-ScR guidelines. Searches were performed in the PubMed, Scopus, Web of Science, Embase, ProQuest, and Dialnet databases covering the period extending from January 2000 to May 2023. Finally, 35 studies were identified for the complete review. A total of 30 studies include variables associated with the structure and process of care, 20 include variables associated with psychological aspects of care, and 23 studies integrate variables related to patient care at the end of life. Analysis suggests that enhancing professional training in aspects such as communication with patients and families, creating intimate spaces with flexible visiting hours, increasing emotional support, promoting frequent contact with healthcare teams, and transparently communicating about illness and imminent death to both families and minors could improve the quality of PPC.

## 1. Introduction

In recent years, significant advances have been made in the technological and medical fields that have helped to extend life expectancy. Situations that used to be a certain cause of death are nowadays survivable and, in fact, often survived [1]. Despite this, thousands of children continue to die every year [2]. An estimate collected by Benini et al. [3] states that at least 10 out of every 10,000 minors are diagnosed with a disease requiring Pediatric Palliative Care (PPC), of whom 1 in 10,000 die annually. Regarding Spain specifically, it is estimated that around 3000 minors die each year and between 5574 and 7432 minors may need PPC [4].

Palliative care (PC) aims to improve the quality of life of patients and their families while life-threatening conditions exist [5,6]. While PC in adults is a well-developed field of care, not all PC-related practices in adults are valid and transcribable for the pediatric population [5]. Consequently, specific quality indicators for PPC must be created. Furthermore, while in the case of adults, PC is mostly intended and set in motion for people with cancer [7], in the case of PPC, it serves minors with a wide variety of diagnoses [6,7], including but not limited to affectations related to neurological, metabolic, and respiratory causes or cardiac diseases [8]. Cancer represents only 4.1% of the total cases of minors treated by PPC [3].

In 2014, the Spanish Ministry of Health, Social Services and Equality published a book entitled “Cuidados Paliativos Pediátricos en el Sistema Nacional de Salud: Criterios de Atención” [4]. The publication includes several aspects that differentiate PPC and its adult-focused counterparts such as a wide plurality of diagnoses, variability in the age of patients, a lower number of cases compared to adults, a limited availability of drugs that can be prescribed to minors, the continuous growth of minors, and a greater social impact throughout the illness, among others.

This, however, may be considered an exception as PC programs for adults exist in almost all European countries while very few have established PPC related programs. In studies such as Davies et al. [9], other barriers were identified regarding PPC such as a lack of resources, lack of training in PPC, and uncertainty regarding prognosis. Additionally, Alonso and Antón [8] and Knapp and Thompson [10] reported on the reluctance of families to use PPC as they perceived such type of care as a surrender.

The purpose of PPC is to provide competent, compassionate, appropriate, and consistent care to those minors diagnosed with a life-limiting illness [11], to reduce their suffering, and to provide opportunities for personal growth. It is recommended that PPC be initiated at the time of diagnosis [3,12] and continued throughout the whole treatment process even while the bereavement period may be taking place [2,6].

The Initiative for Pediatric Palliative Care (IPPC) identified six domains through which to improve the quality of family-centered care: support for the family unit, communication throughout the process, shared decision making with professionals, pain relief, continued care, and bereavement support [13].

In the case of minors, their psychological and emotional needs should also be addressed and will often be tied to their stage of development as well as their friendships and school relationships [14]. Likewise, physical and psychological support for the families should not be forgotten. Pediatric diseases that require PPC tend to modify family dynamics and roles, generating economic and work-related issues, or cause physical and/or social isolation [3].

The theoretical framework in which this study is framed is the “Patient and Family Centered Care” (PFCC) [15] that emerged during the second half of the 20th century. This approach is based on collaboration between professionals, patients, and families through shared decision making to meet the psychosocial and developmental needs of children and the role of families in promoting the health and well-being of their children [15,16]. This entails the need for families and patients to be duly informed and involved throughout the process and for minors to be involved. To do this, it is necessary to create a relationship of trust, respect, and open communication with professionals [15,16]. In this sense, nursing professionals are the ones who dedicate the most time to the care of pediatric patients and therefore who best know the needs of patients and their families. Nursing care is characterized by a holistic approach that covers the patient’s physical, emotional, psychological, and social aspects. Nurses actively engage in the processes mentioned above, provide emotional support, offering resources for grief management, fostering an environment of compassionate care, and empowering families to actively participate in the patient’s care [16].

Therefore, in determining the defining characteristics of PPC, attention is key to being able to inform the development of PPC and, therefore, being able to analyze and propose improvements. Since 2005, the National Consensus Project for Quality Palliative Care (NCP) has published guidance describing eight areas based on the World Health Organization’s definition of PC [17]. In the fourth edition published in 2018, those eight areas are:

1. Structure and process of care;
2. Physical aspects of care;
3. Psychological aspects of care;
4. Social aspects of care;
5. Spiritual, religious, and existential aspects of care;
6. Cultural aspects of care;
7. Care of the patient approaching the end of life;
8. Ethical and legal aspects of care.

There is currently a growing interest in quality of care, and more specifically in quality indicators. Therefore, this scoping review aimed to identify the variables associated with the quality of care of PPC patients in Spain, focusing specifically on three areas: structure and process of care, psychological aspects of care, and care of patients approaching the end of life.

## 2. Materials and Methods

A scoping review of the literature was performed. Scoping reviews aim to offer an overview and summary of the contents related to the topic investigated [18]. This type of review differs from a systematic review because it does not try to evaluate the quality of the results of the included studies but instead tries to analytically reinterpret the literature related to, in this case, PPC and quality of care, and identify research gaps in this area. The scoping review is the most suitable alternative for this topic as it allows all of the different varieties of study designs to be integrated into the study.

The purpose of this scoping review is not to perform a critical appraisal of the results of the studies, but rather to provide an overview and summary of the evidence. Considering that the studies reviewed employ diverse methodological approaches (qualitative and quantitative) as well as diverse techniques (questionnaires, interviews, clinical records review, participant observation, and focus groups), an assessment of methodological quality was not performed, but it was ascertained that the above techniques were adequately used.

To carry out this review, the stages proposed by Arksey and O’Malley [18] were followed: identification of the research question, identification of relevant studies, selection of studies, and data extraction. In addition, the Preferred Reporting Items for Systematic Reviews and Meta-Analyses statement for reporting scoping reviews (PRISMA-ScR) checklist [19] was used to facilitate the presentation and improve the quality of the research. The protocol for this review has not been registered or published.

### 2.1. Identification of the Research Question

The following research question was formulated: “What knowledge exists in the literature about the quality of care regarding PPC in Spain, focusing on the structure and process of care, on psychological aspects of care, and on the care of the patient approaching the end of life?”

### 2.2. Identification of Relevant Studies

An extensive electronic search was performed in the PubMed, Scopus, Web of Science, Embase, ProQuest, and Dialnet databases covering the period extending from January 2000 to May 2023. No additional manual search was performed. Studies were identified by searching for keywords in the titles and abstracts of the articles. The search strategy used in the databases was performed with the following descriptors:

- In English: pediatric palliative care OR ((palliative OR palliative care OR palliative needs OR terminal care OR terminal illness OR end of life) AND (child* OR infan* OR adolesc*)) AND (quality of care OR care quality OR quality care OR quality) AND (Spain OR Spanish);
- In Spanish: Cuidados paliativos pediátricos OR ((paliativo OR atención paliativa OR necesidades paliativas OR cuidados terminales OR enfermedad terminal OR final de la vida) AND (niñ* OR infan* OR adolescen*)) AND (calidad asistencial OR calidad de la asistencia OR calidad de los cuidados OR calidad) AND (españ*).

### 2.3. Selection of Studies

Records identified from the electronic searches were imported into RefWorks to remove duplicates. Titles and/or abstracts were then reviewed and studies relevant to the research question were identified. The full texts of the remaining articles were read and selected based on the criteria listed in Table 1.

### 2.4. Data Extraction

Data extraction from each article was carried out using an Excel spreadsheet including author and year, language, the autonomous community where the research was carried out, methodology and technique used, cohort (patients, families, and/or professionals), and areas that are addressed.

## 3. Results

**Figure 1:**
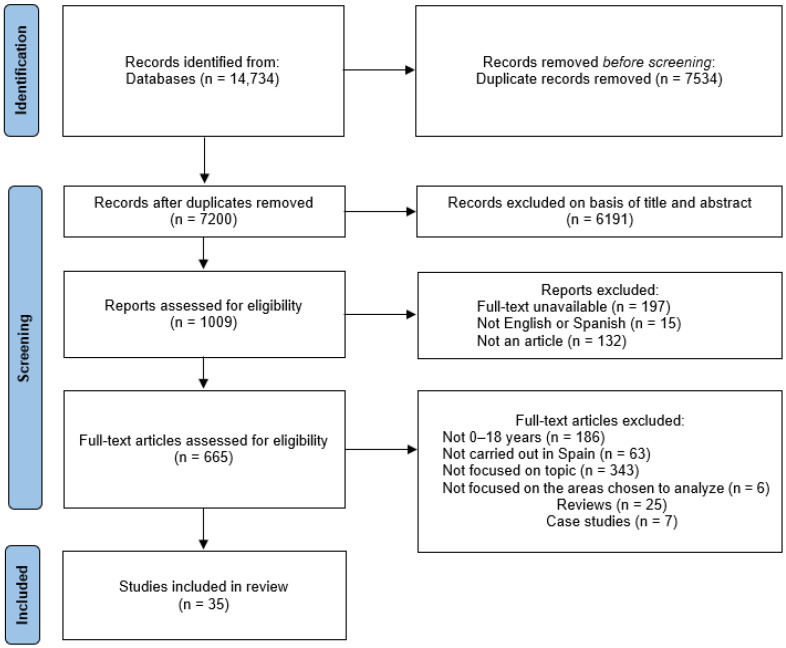
Study selection PRISMA flow chart.

A total of 14,734 records were identified covering the period extending from January 2000 to May 2023. After removing duplicates, 7200 titles and abstracts were reviewed. Articles whose full text was not available were removed (leaving 665 articles), and those that did not meet the inclusion criteria were eliminated. Finally, 35 studies were identified for the complete review. Their main characteristics are presented in Table 2 for data extraction. The study selection process is detailed in the PRISMA flow chart (Figure 1).

If we look at the descriptive characteristics of the articles included, the first striking feature is the number of articles published per year, which shows a growing interest in PPC, going from 7 publications in the period 2000–2009 to 15 between 2010–2019 and 13 from 2020 to May 2023. Regarding language, 60% were published in Spanish and 40% in English.

Regarding the cohorts, almost half of the articles (48.6%) focus on the opinions of professionals, 25.7% on data about patients, 14.3% on the perceptions and opinions of families, 8.6% on the opinions of both families and professionals, and finally 2.9% on patients and families.

Most of the studies were carried out in the Madrid Region (45.7%) and in several centers throughout Spain, thus being multicenter studies (22.9%). Of the remaining studies, 11.4% were carried out in Andalusia, 8.6% in Catalonia, 2.9% in Asturias, 2.9% in Valencia, 2.9% in Extremadura, and another 2.9% in Murcia.

Finally, regarding the source of data collection, questionnaires are the most common (40%), followed by interviews (22.9%), clinical record review (22.9%), and focus groups (2.9%). Other studies combined several techniques such as interviews and field notes (2.9%), interviews and participant observation (2.9%), and clinical records review and questionnaire (5.7%).

The key findings are presented according to three of the eight areas defined by the NCP and differentiated based on the cohorts. Finally, they are summarized in Table 3.

### 3.1. Structure and Process of Care

Of the 35 included studies, 30 include variables associated with the structure of care and care process [20,21,22,23,24,25,26,27,28,29,30,31,32,33,34,35,36,37,38,39,40,41,42,43,44,45,46,47,48,49].

Regarding the care process, the studies related to families [22,26,38,47] as well as patients [37,49] and professionals [20,26,29,41,43,47] include the variable of implementing a personalized care plan from the start of the intervention. 

Most studies point to a lack of professional training [20,23,27,28,29,30,31,33,38,41,42,43,45]. Among the training topics that professionals consider necessary to implement PPC are the use of opioids and pain management [27], the limitation of therapeutic effort (LTE) [41], emotional management [21,26,29,43], the improvement of communication [23,29,31,43,47], and the improvement of the treatment given to families and patients [23,27,47]. Families, for their part, indicate that professionals would need training on LTE [38] and emotional management [26,38,40], as well as training to improve communication [23,47], and to improve the treatment given to families and patients [23,38,47]. Related to training, several studies regarding patients [24,32,34,35,36], but also families [32] and professionals [29,43] address caregiver training/education as a variable that improves the quality of care.

Regarding healthcare teams, 13 studies [20,23,29,33,34,36,37,42,43,44,46,47,48] discuss the need for PPC teams to be made up of professionals from various disciplines including nursing [23,34,36,37,43,47], home care [34,36,37], PPC specialists [20,29,34,36], and other disciplines such as pediatrics [20,34,37,47], psychology [29,33,43], neurology [20,29,34], oncology [29,36], and physiotherapy [34].

In addition, professionals consider team meetings necessary for a better quality of care [28,29,45,47,48], since in several studies both professionals [28,43,46,47] and families [38,47] highlight a lack of team coordination. On the other hand, several patient studies [24,34,37,39,49] add the need to have access to the staff at any time as relevant, a fact with which professionals agree [33,44,48].

Regarding the care environment, families complain about restricted visits [23,40,47]. They also report a lack of privacy [23,25,40,47] and suggest that a place or floor exclusively for PPC [38,47] or individual rooms [40] would significantly improve the quality of care. For their part, professionals also recognize the two problems detected by the families: on one hand, restricted visits [23,30], suggesting more flexible schedules [23,30,43,47], and, on the other hand, the lack of privacy [23,30,47], which would be solved with exclusive spaces for pediatric patients in PC [30,43,47].

Finally, both studies related to families [32,47], as well as patients [32,36] and professionals [47] allude to the possible lack of sufficient resources to care for this segment of the population.

### 3.2. Psychological Aspects of Care

Relating to psychological aspects, 20 studies report variables associated with these aspects [21,22,23,25,26,30,36,38,39,42,43,44,45,47,48,49,50,51,52,53].

Regarding the variable of bereavement, the need for attention and support for families is expressed not only by the families themselves [23,25,38,47] but also by professionals [23,43,47] and patients [39]. There is also a need for emotional support for the care team so that they can carry out their work with the best possible quality. This variable is mentioned above in the studies regarding professionals [21,23,30,42,43,47,48,50] but also families [22,23,47]. The most mentioned items are evaluating the distress and bereavement of professionals [42,48], relieving stress [21,44,47,52] and exhaustion [21,44,52], preventing burnout [21,42], and the need for strategies to cope with death [21,30,42,45] such as the one used in two studies consisting of depersonalizing patients [21,52].

Families demand greater referral to psychological care not only during bereavement but throughout the entire care process [23,25,38,47] to learn how to deal with family conflicts and cope with the disease [22,38,47], as well as support to cope with uncertainty, emotional or existential distress [22,26,38,47,53], family support for care burden [25], and education about the disease, its symptoms, treatment, and aftereffects [47,50,53].

Professionals also explain the need to refer families to psychological care [23,47], to offer them support to avoid caregiver overload [44], and to confront feelings of distress and uncertainty [26,30,43,47,48]. In studies regarding patients, the needs that are collected are mainly related to family support for avoiding compassion fatigue [36] and education about the disease [36,49] to cope with it [51].

Concerning the need or not to talk about death, only two patient studies affirm that it is necessary [36,51], while families [22,25,26,38] and professionals [26,30] consider that it is not necessary to address this topic with children or adolescents.

Finally, another variable mentioned within this area is recognizing and treating psychological problems. Among those most frequently mentioned by families and professionals are anxiety, stress, helplessness, frustration, sadness, apathy, and anger [22,26].

### 3.3. Caring for the Patient Approaching the End of Life

Lastly, 23 studies integrate variables related to patient care at the end of life [20,23,24,25,27,29,30,31,33,34,35,36,37,38,39,40,43,46,47,48,49,50,54].

Among the variables that patients mention in the studies as relevant to the quality of their care are being told about signs and symptoms [24,37], being able to talk about impending death, and maintaining frequent contact with the care team in the days before [49,54]. Furthermore, patient studies also mention the presence of the care team at the time of death [24,34], post-death care by the team with the families [36,39], and the performance of autopsies and/or organ donations [37,54]. 

Meanwhile, families attach importance not only to post-death care [25,40,47] and communication of signs and symptoms [23,25,38], but they also value keeping memories of their children [25,47], the performance of farewell rituals, and the presence of the care team at the funeral [25]. However, they mention the lack of information before the time of death as a negative aspect of the care received [38,50].

Professionals agree with families and patients on aspects already mentioned such as the importance of communicating signs and symptoms [23,29], frequent contact in the previous days [30], talking about imminent death [27,29,30,46], being present at the time of death of the child [30,46,47], providing post-death care to families [29,47] and helping them in autopsy and/or donation processes [30]. Like families, professionals consider that sometimes there may be a lack of information given to families in moments before death [48,50].

Regarding the place to wait for death, most studies that addressed this issue stated that both home and the hospital were valid places [20,24,25,29,33,35,36,39,40]. Only two studies stated that the best place to pass away was home [31,34]. Related to this, studies of patients [39,49], families [25], and professionals [29] reported the need to evaluate patients’ preferences regarding the place to die. At an ethical level, it is imperative to ensure understanding and respect for the child’s rights and wishes, always adapting information to their level of comprehension and considering their ability to express their preferences in care [48,50].

Other studies also recognized as important the patients’ preferences regarding visits at the end of life [23,30,43,54]. Thus, if the chance to say goodbye or to be accompanied at the moment of death is evaluated, families [23,25,38,40,47], patients [24,34,54], and professionals [23,30,43,47] agree on the importance of this fact.

## 4. Discussion

This review contributes to expanding knowledge about the current state of PPC in Spain and about the variables that patients, families, and professionals associated with the quality of care in aspects related to the structure and process of care, psychological aspects of care, and the care of the patient approaching the end of life.

On the one hand, the findings of this scoping review confirm that regarding PPC, the greatest need currently encountered is the lack of training of professionals. To maintain good care, it is necessary to have continuous training to allow the acquisition of the knowledge for identifying children in need of PPC and evaluating their needs [4]. The development of skills to alleviate the physical, psychological, spiritual, and social suffering of the child and their families as much as possible is also key [4]. Several studies affirm that between 13.9% and 55% of healthcare professionals consider that they are not prepared to care for a patient in need of PC [20,30,31], but almost all (more than 80%) would be willing to receive training [20,27,30,31]. This reflects the predisposition of health professionals to improve their care of PPC patients, which is often based on personal resources and previous experience since specific training in this area is often insufficient [31]. The substantial demand for care aimed at critically ill pediatric patients underlines the importance of having the necessary knowledge, skills, and attitudes to provide them with appropriate care [55] as well as foster fluid collaboration between different levels of care and contribute to the establishment of a high-quality network, thus enabling a more comprehensive, unified, coordinated, and multidisciplinary approach that improves the quality of life of patients and their families [28].

On the other hand, the analysis shows that the quality of care could be improved if visiting hours were made more flexible and more intimate spaces were used. The length of stay allowed for accompanying persons in Spain varies between 1 h and 24 h depending on the hospital [30]. However, in most of them, families cannot accompany minors for the whole day, even though the European Charter for Children in Hospital [56] recognizes their right to be accompanied by their families for as long as possible. In addition, the visiting regulations do not allow access to hospitals for children under the age of 12, so siblings do not have the option of visiting. Although it is said that PPC should take care of the family and patient as a whole, few studies address the needs of siblings. It would be convenient to carry out more research that evaluates these needs and includes details on how the siblings experience the process. Regarding spaces reserved for PPC, the works of Bosquet-del Moral et al. [38], Tagarro García and Ruza Tarrió [40], and Casanueva-Mateos et al. [47] underline the need for families to have adequate and more defined spaces. Both individual rooms and an exclusive floor for PPC are suggested as great alternatives to achieve greater privacy, thus improving the quality of care. However, this may not be possible since not all provinces have specific services for PPC, thus generating inequities [57]. The increase in the number of patients requiring PPC represents a high demand for resources for the National Health System, and it is a challenge to ensure that these resources are available to everyone, regardless of their geographic location [32]. 

Regarding emotional support, the need to increase it to deal with situations with poor prognosis for minors is highlighted. In addition, families consider that psychological support services should be extended not only during the disease process but also after death in the bereavement process [38]. Although some studies indicate a request by families to include psychology professionals in the care teams [43], other families state that they prefer to be listened to with empathy and only 29% would like professional psychological support [23].

In addition to families, there is also demand for psychological support from healthcare teams. A study by Lledó-Morera and Bosch-Alcaraz [42] states that 25.49% of nurses express the importance of having an environment in which they can share their feelings and emotions experienced following a patient’s death. The ability to face and assimilate these situations often depends on the personal resources of the professionals, especially when they do not have specific training [30]. Having coping strategies can provide better assistance to the patient and family.

Relative to the care of the patient approaching the end of life, the place of death is an important factor that is often used as a quality indicator in the evaluation of PPC [49]. The choice of where to wait for death, whether in the hospital or at home, is often conditioned by factors such as the training the families have received to take care of the child, the suitability of the home, the availability of the team 24 h a day, or the severity of the child’s situation [33]. Although many families express a preference for the death of their child to occur at home (56% according to Peláez-Cantero et al. [49])—and between 77% and 97% of professionals agree [20,31,33]—the importance of the individualization of each case must be recognized. It would be interesting to study the possibility of creating intermediate settings that could provide a less clinical environment than a hospital and more support than the home, for those cases in which this option is appropriate.

However, and related to the above stated, when death occurs in the hospital, medical teams consider it important to be present and accompany the family at that moment [30,34]. Families value very positively the moments that occur after death, the immediate care, the treatment of the body [40], and being able to keep memories such as the umbilical cord clamp or the identification bracelet among others [25].

In addition, allowing the family to remain at the bedside at the time of death stands out as a relevant variable related to the quality of care. It is considered as something positive by 64% of families and 40% of professionals according to Tagarro García et al. [23]. Other studies also indicate the importance of family and friends being able to say goodbye [25,30,38,40].

### Limitations of the Study

This scoping review was based on a systematic approach and evaluated a wide variety of studies covering three different perspectives (patients, families, and the professional) in three different areas (structure and process of care, psychological aspects of care, and care of the patient approaching the end of life). This approach provided a better understanding of the needs in the field of PPC. However, it is important to mention that this review has certain limitations. First, the inclusion was restricted to studies published in English or Spanish, which could have resulted in the omission of relevant research in other languages. Furthermore, a manual search of key journals was not carried out, and gray literature was not considered, as six common databases were searched. Importantly, although this review is not intended to establish definitive conclusions about all the needs analyzed, it provides a valuable contribution to the evidence base on PPC in Spain and provides the basis for future research in this field.

## 5. Conclusions

This study, by identifying and addressing the specific needs of patients in CPP, has the potential to generate substantial improvements in different aspects. First, the results obtained could translate into tangible improvements in the quality of life of patients, providing them with more effective care adapted to their needs. In addition, facilities for the families of admitted children should be included, such as frequent contact with the care team, flexible visiting hours, availability of individual rooms for greater privacy, and bereavement support, also considering the needs of siblings. This direct improvement not only benefits the children and their families, but also has a positive impact on society in general, which could potentially improve their perception of PPC.

Furthermore, the results may contribute to the optimization of healthcare resources by identifying more precisely the specific demands of pediatric patients regarding PC. These results underline the need to make significant changes in healthcare structures at the institutional level to improve the quality of care and establish more specialized care environments for pediatric patients requiring PC. In particular, it is important to train healthcare teams in pediatrics as well as in other services.

Finally, the study results not only have immediate implications but also lay the foundation for future research about the effectiveness of services, quality of care, and the formulation of suitable policies. This ongoing progress contributes to the advancement of knowledge and the continual enhancement of care practices and medical breakthroughs in the field of PPC, benefiting future generations of pediatric patients and their families.

## Figures and Tables

**Table 1:** Inclusion and exclusion criteria.

Inclusion Criteria	Exclusion Criteria
Studies published between 2000 and 2023 In English and Spanish Full text available Patients between 0 and 18 years old Carried out in Spain Qualitative, quantitative, and mixed methods studies published in scientific journals Studies that collect variables on the structure and process of care, on psychological aspects of care, and on the care of the patient approaching the end of life	Reviews and case studies Books, book chapters, doctoral theses, congress abstracts, letters, editorials, commentaries Studies that are not focused on PC Studies where care is not linked to the hospital setting

**Table 2:** The main characteristics of the included studies.

Author/s	Year	Language	Region	Aim	Main Technique	Cohort	Area/s Addressed
Calleja Gero et al. [20]	2011	SPA	Madrid	Assess the knowledge of, and interest and involvement in, PPC among Spanish pediatric neurologists	Questionnaires	Prof.	Area 1 Area 3
Rodríguez-Rey et al. [21]	2019	ENG	SPAIN (Madrid, Basque Country, Murcia, Asturias, Castile-Leon)	Explore the prevalence of burnout syndrome (BOS) and post-traumatic stress disorder (PTSD) in a sample of Spanish staff working in nine pediatric intensive care units (PICUs) and compare these rates with a sample of general pediatric staff and explore how resilience, coping strategies, and professional and demographic variables influence BOS and PTSD	Questionnaires	Prof.	Area 1 Area 2
Montoya-Juárez et al. [22]	2013	ENG	Andalusia	Determine the elements parents identify their hospitalized children as suffering, and establish observational indicators for the detection and the interpretation of suffering in children necessary for evaluation and personalized intervention by professionals	Interviews	Fam.	Area 1 Area 2
Tagarro García et al. [23]	2008	SPA	Madrid	Evaluate end-of-life care in a PICU	Questionnaires	Prof. + Fam.	Area 1 Area 2 Area 3
García-Salido et al. [24]	2018	SPA	Madrid	Understand the characteristics of the patients referred from the PICU to the PPCU during the study period	Clinical records review	Pat.	Area 1 Area 2 Area 3
Plaza Fornieles et al. [25]	2020	SPA	Murcia	Evaluate whether PPC team interventions improve end-of-life care, according to parents’ experiences and degrees of satisfaction with the care received	Questionnaires	Fam.	Area 1 Area 2 Area 3
Montoya-Juárez et al. [26]	2012	SPA	Andalusia	Determine, through the discourse of parents and professionals, the signs of suffering evidenced by ill pediatric inpatients and establish observational indicators	Interviews	Prof. + Fam.	Area 1 Area 2
Caballero Pérez et al. [27]	2018	SPA	SPAIN (all regions except Castile-Leon)	Assess the impact of the availability of specialized PPCUs in the management of children eligible for palliative care by their Primary Care pediatricians	Questionnaires	Prof.	Area 1 Area 3
Morán Roldán and García-Mauriño [28]	2021	SPA	Madrid	Analyze the current and global situation of PPC in the area of Primary Care in the Community of Madrid from the point of view of the PC pediatricians themselves	Questionnaires	Prof.	Area 1
Gutiérrez Rada and Ciprés Roig [29]	2020	SPA	Catalonia	Explore the experiences of referring professionals of patients who died while being treated by a PPCU	Interviews	Prof.	Area 1 Area 3
Martino Alba et al. [30]	2007	SPA	SPAIN (regions not indicated)	Determine how often pediatric intensivists have to manage dying patients, their approach to these patients, their knowledge of this field, and their needs	Questionnaires	Prof.	Area 1 Area 2 Area 3
Moya-Dionisio [31]	2020	SPA	Asturias	Understand and assess the experiences of and knowledge among Primary Care pediatricians about PPC in the Principality of Asturias (Spain)	Questionnaires	Prof.	Area 1 Area 3
Pérez-Ardanaz et al. [32]	2019	ENG	Andalusia	Analyze the use of health services for children with severe chronic diseases, seeking to identify patterns of use according to sociodemographic and clinical conditions, and identify unmet needs of care coordination that could benefit from nursing case management services	Clinical records review and questionnaires	Pat. + Fam.	Area 1
Astray San Martín [33]	2010	SPA	Madrid	Assess the knowledge among Primary Care pediatricians in Area 5 of Madrid of PPC and their possible involvement in it	Questionnaires	Prof.	Area 1 Area 3
García-Salido et al. [34]	2015	ENG	Madrid	Describe the clinical evolution and needs of children with spinal muscular atrophy type I treated in a domiciliary palliative care program	Clinical records review	Pat.	Area 1 Area 3
De Noriega et al. [35]	2022a	ENG	Madrid	Describe the characteristics of cancer patients and healthcare delivered by a PPCU with a home hospitalization program	Clinical records review	Pat.	Area 1 Area 3
De Noriega et al. [36]	2022b	ENG	Madrid	Describe the attention provided by a PPC team to patients with CNS cancer and the differences in care compared to patients who did not receive PPC	Clinical records review	Pat.	Area 1 Area 2 Area 3
Bobillo-Pérez et al. [37]	2020	ENG	Catalonia	Describe how end-of-life care is managed when life-support limitation is decided in a PICU and analyze the influence of the further development of the Palliative Care Unit	Clinical records review	Pat.	Area 1 Area 3
Bosquet-del Moral et al. [38]	2012	SPA	Andalusia	Determine the experiences of grieving in mothers who suffered the loss of their child after a cancer disease process	Interviews	Fam.	Area 1 Area 2 Area 3
Fernández Navarro et al. [39]	2000	SPA	Valencia	Presentation of the care activity carried out in the first 17 months of operation of the home hospitalization program of the La Fe Children’s University Hospital in Valencia	Clinical records review	Pat.	Area 1 Area 2 Area 3
Tagarro García and Ruza Tarrió [40]	2008	SPA	Madrid	Understand in depth parental perceptions of potential improvements relating to end-of-life care in the PICU	Interviews	Fam.	Area 1 Area 3
García Caballero et al. [41]	2020	SPA	SPAIN (regions not indicated)	Ascertain whether internists know what limitation of therapeutic effort (LTE) means and whether training in palliative care affects this understanding	Questionnaires	Prof.	Area 1
Lledó-Morera and Bosch-Alcaraz [42]	2021	ENG	Catalonia	Evaluate how nurses cope with the death of a pediatric patient, relate it to the different sociodemographic variables, and describe personal coping strategies used by nurses in managing the process and accepting the death of the patient	Questionnaires	Prof.	Area 1 Area 2
González-Gil et al. [43]	2021	ENG	Madrid	Explore nurses’ experiences related to promoting the visits of siblings to the PICU	Interviews	Prof.	Area 1 Area 2 Area 3
Mota Vargas et al. [44]	2016	ENG	Extremadura	Analyze the professional trajectory of palliative care workers over time and the factors which influence this trajectory	Interviews	Prof.	Area 1 Area 2
González-Gil [45]	2008	SPA	Madrid	Analyze the conceptualization nurses have of child death in a PICU and identify sociocultural strategies for coping with it	Interviews and participant observation	Prof.	Area 1 Area 2
García-Salido et al. [46]	2016	SPA	SPAIN (regions not indicated)	Primary objective: describe the management and monitorization of critically ill pediatric hemato-oncology patients (CIPHOs) in the Spanish PICU. Secondary objective: through a literature review, identify possible areas of improvement	Questionnaires	Prof.	Area 1 Area 3
Casanueva-Mateos et al. [47]	2007	SPA	Madrid	Gain greater insight into the experiences of the parents of children admitted to the PICU and of the health workers in these units	Focus groups	Fam. + Prof.	Area 1 Area 2 Area 3
Rico-Mena et al. [48]	2023	ENG	Madrid	Describe the feelings and emotions of professionals working in an interdisciplinary pediatric palliative home care team	Interviews and field notes	Prof.	Area 1 Area 2 Area 3
Peláez-Cantero et al. [49]	2023	ENG	SPAIN (Catalonia, Madrid, Andalusia, Balearic Islands, Basque Country, Aragon, Murcia, Castile-La Mancha, Valencia, and Canary Islands	Analyze the characteristics of patients who die in the care of specific PPC teams	Clinical records review and questionnaires	Pat.	Area 1 Area 2 Area 3
Pascual-Fernández [50]	2014	SPA	Madrid	Describe the aspects of information offered to relatives of patients in the end-of-life process in Intensive Care Units (ICUs), determine nursing evaluation in this process, and evaluate professionals’ attitude on this subject	Questionnaires	Prof.	Area 2 Area 3
Hernández Núñez-Polo et al. [51]	2008	SPA	Madrid	Analyze the information that children with terminal cancer have, as well as the degree of communication between parents and children about death, and describe the variables that can influence the patient’s information	Clinical records review	Pat.	Area 2
Pérez-Ardanaz et al. [52]	2022	ENG	SPAIN (regions not indicated)	Determine the quality of working life among different pediatric nursing professionals, taking into account sociodemographic and work context factors, and the relationships of burnout, compassion satisfaction, and compassion fatigue between different job positions	Questionnaires	Prof.	Area 2
Rico-Mena et al. [53]	2019	ENG	Madrid	Explore parents’ experiences regarding the implementation of a physical rehabilitation program in PPC	Interviews	Fam.	Area 2
Agra Tuñas et al. [54]	2019	SPA	SPAIN (regions not indicated)	Describe the different types of child deaths in the PICU in Spain, and analyze the characteristics of those dying from an LTE	Clinical records review	Pat.	Area 3

Note: SPA = Spanish; ENG = English: Prof. = Professionals; Fam. = Families; Pat. = Patients; Area 1 = Structure and process of care; Area 2 = Psychological aspects of care; Area 3 = Caring for the patient approaching the end of life.

**Table 3:** Key findings of the results.

	Aspect	Key Findings
Structure and Process of Care	Professional Training	Lack of training identified in areas like opioid use, pain management, emotional management, and communication
	Interdisciplinary Teams	Emphasis on the need for diverse teams and team meetings for better quality care
	Care Environment	Concerns about restricted visits, lack of privacy, and a call for dedicated spaces for PPC
	Resource Availability	Studies indicate a potential lack of sufficient resources for this segment of the population
Psychological Aspects of Care	Bereavement Support	Families, professionals, and patients express the need for attention and support, both during and after bereavement
	Emotional Care for Care Team	Professionals emphasize the importance of emotional support, coping strategies, and stress relief in preventing burnout
	Psychological Care for Families	Demand for greater referral to psychological care throughout the entire care process, addressing family conflicts and more
	Preferences Regarding Death Discussions	Varied opinions on discussing death with children or adolescents
	Recognition of Psychological Problems	Anxiety, stress, helplessness, and other psychological problems were commonly mentioned by families and professionals
Caring for the Patient Approaching the End of Life	Patient Preferences	Patients value being informed about signs, discussing impending death, maintaining contact with the care team, and post-death care
	Family Preferences	Families emphasize post-death care, communication of signs and symptoms, preserving memories, and the importance of saying goodbye
	Professional Perspective	Professionals agree on the importance of communicating signs and symptoms, discussing imminent death, being present at the time of death, and providing post-death care
	Place of Death	Varied opinions on the best place to pass away
	Ethical Considerations Targeting the Children	Adapt information to the child’s comprehension, respecting rights and wishes (always considering the child’s ability to express preferences)
	Saying Goodbye and Accompaniment	Universally recognized as crucial by families, patients, and professionals

## Data Availability

Not applicable.
